# A cannabinoid-intoxicated child treated with dexmedetomidine: a case report

**DOI:** 10.1186/s13256-015-0636-2

**Published:** 2015-07-03

**Authors:** Flora Cipriani, Aldo Mancino, Silvia Maria Pulitanò, Marco Piastra, Giorgio Conti

**Affiliations:** Pediatric Intensive Care Unit, Department of Intensive Care and Anesthesia, Agosto Gemelli University Polyclinic, Catholic University of Rome, Largo Agostino Gemelli 1, 00168 Rome, Italy

**Keywords:** Cannabinoids, Dexmedetomidine, Pediatric intensive care

## Abstract

**Introduction:**

In the last 20 years, the rate of exposure to marijuana has increased dramatically, even in the pediatric population. Effects of intoxication are variable, more severe neurological symptoms can be observed following ingestion, thus hospital or intensive care unit admission is often required. Usually cannabinoids intoxicated patients are treated with administration of benzodiazepines or opioids, accepting the related risk of intubation and mechanical ventilation. Dexmedetomidine is a highly selective α_2_-adrenergic receptor agonist, with no effect on the respiratory drive and pattern and produces a good level of sedation, allowing to avoid the administration of other sedatives. To our knowledge, this is the first reported case of dexmedetomidine use to support a cannabis intoxicated patient.

**Case presentation:**

A 19-month-old Caucasian boy was presented to our emergency department. At the time of his arrival, he was somnolent with paroxysms of agitation, breathing spontaneously and hemodynamically stable. The results of all investigations were negative, but the result of the immunochemical screening of his urine was positive for Δ^9^-tetrahydrocannabinol. The patient was admitted to the pediatric intensive care unit and treated with a continuous infusion of dexmedetomidine.

**Conclusions:**

Dexmedetomidine is a fairly safe and effective antidote for pediatric marijuana or natural cannabinoid exposures. Its properties and potential to allow for “cooperative” sedation make it a more attractive choice with fewer side effects than benzodiazepines or opioids.

## Introduction

In the last 20 years, a progressively higher prevalence of cannabis consumption has been detected. Thus, the rate of exposure has increased dramatically, even in the pediatric population. Historically, significant effects following unintentional pediatric marijuana ingestion were rare, and the relevant literature consists of single case reports and a small case series [1–5]. In the United States, the decriminalization of medical marijuana has resulted in a significant increase in marijuana exposures in young children, and more severe symptoms can be observed, owing to the higher concentrations of marijuana in edible and medical products [6, 7].

Accidental consumption is mostly oral among children, and effects are variable, with slow onset and prolonged duration. Symptoms are mostly neurological and range from lethargy to deep coma [1–3], with periods of agitation and psychotic reactions, dysphoric screaming and posturing; seizures are rare [3]. Possible non-neurological symptoms are bradypnea and apnea, mydriasis, hyporeflexia and hypotonia, conjunctival hyperemia, tremor, emesis and tachycardia.

Ingested marijuana has an absorption of 5% to 10 % and results in effects 1 hour after consumption. Δ^9^-tetrahydrocannabinol (THC) is the primary psychoactive component of marijuana. It reaches peak plasma concentrations after 1–6 hours, and its plasma levels remain elevated, with effects lasting up to 5–24 hours. Marijuana is metabolized in the liver, and the metabolites are excreted in urine. Urinary metabolites can be detected up to 12 days after consumption [5].

Management of acute marijuana intoxication is primarily supportive. Benzodiazepines may be considered to control panic attacks and agitation [4]. Children should be observed for at least 6 hours after ingestion, and for 24 hours if symptomatic because THC has a terminal half-life of 20–30 hours [8].

In this report, we describe a pediatric case of accidental cannabinoid intoxication treated with dexmedetomidine to support the patient, thereby avoiding cardiovascular instability and the risk of mechanical ventilation.

## Case presentation

A 19-month-old Caucasian boy was presented to our emergency department in the morning. At the time of arrival, he was lethargic, responsive to painful stimulus with jarring cry, and with isocoric and isocyclic midpoint pupils. He was breathing spontaneously on room air with peripheral saturation of oxygen (SpO_2_) around 96 % and periods of apnea, lasting around 5 seconds, that were not associated with bradycardia. Hemodynamically, he was stable (systolic blood pressure: 125mmHg; heart rate: 160 beats per minute). The patient was somnolent with paroxysms of agitation. The mother reported that the somnolence had developed about 12 hours before presentation, after the child had eaten something he had found on the ground at a park. Emergency brain computed tomography showed no vascular, bone or parenchymal alterations. Hematological and blood chemistry investigations were normal. Immunochemical screening of his urine was performed, and the results were negative for everything (amphetamines, barbiturates, benzodiazepines, cocaine, methadone, opiates and tricyclic anti-depressants) but natural cannabinoids (THC); synthetic cannabinoids were not tested. Subsequently, we collected serial blood samples for quantitative testing and a hair sample to evaluate chronic cannabis exposure.

**Fig. 1:**
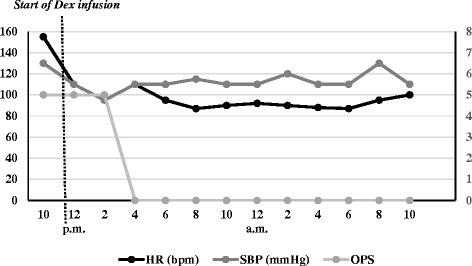
Variation of heart rate (HR), systolic blood pressure (SBP) and Objective Pain Scale (OPS) score over time. The figure shows the trend of the patient’s vital signs (HR and SBP), which decreased after the start of the dexmedetomidine (Dex) continuous infusion (at 12 p.m.) and later remained stable. The graph also shows a zeroing of the OPS score because the child stayed quiet a few hours after the beginning of dexmedetomidine treatment. bpm, Beats per minute

We admitted the patient to our pediatric intensive care unit (PICU) for monitoring and supportive treatment. The child had normal vital signs and was resting quietly for most of the time; however, these periods of calm were interrupted by sudden-onset agitated outbursts of violent behavior. First, we tried to control these phases with non-pharmacological measures: quiet ambience in the room, toys and colors, music and videos, presence of parents and gentle wrapping of the child in the bed. At a later stage, we tried to control his agitation by administering an intravenous bolus of midazolam (1mg), which was followed by a brief obstructive apnea episode. Considering our experience with dexmedetomidine and knowing it has no depressive respiratory effect [9], we started a dexmedetomidine continuous infusion, without any bolus, at the rate of 0.4μg/kg/hr and increased the rate progressively up to 0.7μg/kg/hr. The clinical effects are shown in Table 1 and Fig. 1. The patient’s episode of agitation disappeared with the dexmedetomidine continuous infusion, and he was quiet and cooperative. His vital sign parameters stabilized (Fig. 1), and his SpO_2_ stayed around 97 % to 100 % with spontaneous breathing on room air. After a 24-hour infusion, we progressively reduced dexmedetomidine until it was stopped while observing the patient’s reaction. He was calm, had a full recovery of consciousness and his vital signs were stable. The patient was transferred to the pediatric general ward.

**Table 1:** Objective Pain Scale (OPS)

Parameter	Finding	OPS score at 11:00 a.m.	OPS score at 4:00 p.m.
Crying	0: Not crying	2	0
	1: Responds to tender loving care		
	2: Does not respond to tender loving care		
Movement	0: No movements	1	0
	1: Restless		
	2: Thrashing or rigid		
Agitation	0: Asleep or calm	1	0
	1: Mild agitation		
	2: Hysterical		
Systolic blood pressure (compared with normal values)	0: Increased <10%	1	0
	1: Increased 10–20%		
	2: Increased >20%		

Specific distress behaviors associated with pain are helpful in quantifying pain or agitation in children unable to provide self-reported information. Several behavioral scales are available. The OPS is one of them and is commonly used in clinical practice. This scale incorporates four pain behaviors (crying, movement, agitation and verbalization) and blood pressure variation, a physiological measure of pain. Each of these categories is scored from 0 to 2, with a score ≥6 indicating intense pain

## Discussion

To the best of our knowledge, this is the first reported case of a pediatric patient with cannabinoid intoxication who was treated with dexmedetomidine. In the reported case, the use of dexmedetomidine allowed us to control the patient’s phases of agitation and aggressiveness without administering benzodiazepines or opioids, thereby avoiding any delirium or respiratory depression and thus the risk of intubation, mechanical ventilation and the need of further sedation. It was possible to evaluate the state of consciousness of the child and to move him out of our PICU only 24 hours after admission.

Dexmedetomidine was approved by the U.S. Food and Drug Administration in 1999 in the United States for use only as a short-term medication (<24 hours) for analgesia and sedation in the intensive care unit (ICU). Its unique properties render it suitable for sedation and analgesia during the whole peri-operative period: as a pre-medication, as an anesthetic adjunct for general and regional anesthesia and as a post-operative sedative and analgesic. It is widely used in the pediatric setting for sedation in ICU, as an analgesic after surgery, in burn patients who are inadequately sedated with opioids or benzodiazepines, for sedation in both non-invasive (i.e., infants diagnostic radiologic procedures) and invasive (i.e., placement of central venous lines, bronchoscopy, laryngoscopy, cardiac catheterization) procedures, in the post-anesthesia care unit to decrease the incidence of agitation, and to perform awake craniotomies [10].

Dexmedetomidine is an imidazole compound and a highly selective α_2_-adrenergic receptor agonist. These receptors are pre- and post-synaptic. Through a negative feedback mechanism, pre-synaptic α_2_-adrenergic receptors inhibit the release of norepinephrine, terminating the propagation of pain signals.

Dexmedetomidine has sedative and analgesic properties. Both supraspinal and spinal sites modulate the transmission of nociceptive signals in the central nervous system, and even peripheral α_2_-adrenergic receptors may mediate anti-nociception. At the spinal level, dexmedetomidine produces its analgesic effects by acting on the α_2_-adrenergic receptors in the dorsal horns of the spinal cord; thus, the drug inhibits the firing of nociceptive neurons and modulates the release of substance P. Dexmedetomidine acts even at a central level. The locus coeruleus is an important modulator of vigilance and nociceptive neurotransmission and has a high density of α_2_-adrenergic receptors; therefore, the major sedative, anxiolytic and even anti-nociceptive effects of dexmedetomidine result primarily from its activity on this site.

The sedation produced by dexmedetomidine is different from that produced by GABAergic drugs (benzodiazepines and propofol). Patients receiving dexmedetomidine infusions have been described as being very easy to wake up and having the ability to follow commands and cooperate; undisturbed, patients were noted to fall asleep right away. This is called “cooperative sedation” [11]. Moreover, the α_2_-adrenergic agonists also act through the endogenous sleep-promoting pathways to exert their sedative effect [12]. There is no respiratory depression and limited cardiovascular effects, providing wide safety margins.

The half-life of dexmedetomidine is around 2 hours, with a rapid distribution phase and a half-life of distribution of approximately 6 minutes. No significant sex- or age-based differences in the pharmacokinetic profile have been reported, even in patients with renal failure. Therefore, dexmedetomidine does not tend to accumulate, in contrast to other agents, such as benzodiazepines and opioids, which may have an unpredictable and prolonged duration of action, especially in critically ill patients [13].

Post-synaptic activation of α_2_-adrenergic receptors in the central nervous system inhibits sympathetic activity and thus can cause bradycardia and hypotension. Usually, these temporary effects are successfully treated with atropine or ephedrine and volume infusions. Often, they occur during or briefly after the loading dose; thus, they can be reduced by omitting or reducing it. Additionally, hypertension may occur during the loading dose or with high infusion rates [14].

Several studies showed that dexmedetomidine might enhance patient safety and comfort in long-term sedation. It reduced the incidence of coma and delirium compared with lorazepam [15]; it reduced duration of mechanical ventilation and delirium; and, compared with midazolam and propofol, it improved patients’ ability to communicate about their pain [15].

## Conclusions

Dexmedetomidine is a fairly safe and effective antidote for pediatric marijuana or natural cannabinoid exposures. Its properties and potential to allow for “cooperative” sedation make it a more attractive choice with fewer side effects than benzodiazepines or opioids.

## Consent

Written informed consent was obtained from the patient’s parents for publication of this case report and any accompanying images. A copy of the written consent is available for review by the Editor-in-Chief of this journal.

## Abbreviations

bpm: Beats per minute
Dex: Dexmedetomidine
GABA: γ-Aminobutyric acid
ICU: Intensive care unit
OPS: Objective Pain Scale
PICU: Pediatric intensive care unit
SpO_2_: Peripheral saturation of oxygen
THC: Δ^9^-tetrahydrocannabinol
